# Birth weight differences between preterm stillbirths and live births: analysis of population-based studies from the U.S. and Sweden

**DOI:** 10.1186/1471-2393-12-119

**Published:** 2012-10-30

**Authors:** Xun Zhang, KS Joseph, Sven Cnattingius, Michael S Kramer

**Affiliations:** 1 Departments of Pediatrics, McGill University Faculty of Medicine, Montreal, Canada; 2 Department of Epidemiology, Biostatistics and Occupational Health, McGill University Faculty of Medicine, Montreal, Canada; 3 Department of Obstetrics & Gynaecology, Women’s Hospital and Health Centre of British Columbia, and School of Population and Public Health, University of British Columbia, Vancouver, Canada; 4 Clinical Epidemiology Unit, Department of Medicine, Karolinska Institutet, Stockholm, Sweden

**Keywords:** Stillbirth, Preterm birth, Fetal growth, Intrauterine growth restriction

## Abstract

**Background:**

Many stillbirths show evidence of fetal growth restriction, and most occur at preterm gestational age. The objective of this study is to compare birth weights at preterm gestational ages between live births and stillbirths, and between those occurring before or during labour.

**Methods:**

Based on singleton births from the United States (U.S.) 2003–2005 (n=902,491) and Sweden 1992–2001 (n=946,343), we compared birth weights between singleton live births and stillbirths at 24–36 completed weeks of gestation from the U.S. and at 28–42 completed weeks from Sweden.

**Results:**

In both the U.S. and Sweden, stillbirth weight-for-gestational-age z-scores were at least one standard deviation lower than live birth z-scores at all preterm gestational ages (GA). In Sweden, no birth weight difference was observed between antepartum and intrapartum stillbirths at preterm GAs, whereas birth weights among intrapartum stillbirths were similar to those among live births at 37–42 weeks.

**Conclusions:**

Birth weights observed at preterm gestation are abnormal, but preterm stillbirths appear to be more growth-restricted than preterm live birth. Similar birth weights among ante- and intrapartum preterm stillbirths suggest serious fetal compromise before the onset of labor.

## Background

Depending on jurisdiction, stillbirth is variably defined as a fetal death from 20, 22, 24 or 28 completed weeks of gestation and can also depend on birth weight [1-4]. In the United States, stillbirth rates have decreased only modestly over the past 20 years (from 7.5 per 1,000 in 1990 to 6.2 per 1,000 in 2005) [3,5,6], while infant mortality has fallen by more than 30% over the same time period. Thus, stillbirth now accounts for over 50% of all perinatal deaths; it remains a significant and understudied problem [3,5]. The causes of a large fraction of stillbirths remain unknown, even when extensive testing and autopsy are performed [7-11]. Classification of causes of stillbirth is often difficult and ambiguous because of the “invisible” nature of the events leading to death in utero. Many systems have been developed for classifying causes of stillbirth, but none has been universally accepted [10,11].

The actual time of fetal death is usually unknown. It has been reported that stillbirths are generally delivered within 24 hours of diagnosis [12-14], and the date of death for antepartum stillbirths is often arbitrarily set as 2 days before delivery [15,16]. Many stillbirths show evidence of fetal growth restriction, and most occur at preterm gestational ages [17-20]. However, birth weights even among live-born preterm infants are known to be lower than fetal weights of their counterparts who remain in utero at the same gestational age [21,22]. Our objective in this study was to compare the birth weights between preterm stillbirths and live births, and between those occurring before or during labour.

## Methods

### U.S. births

We used period linked birth-infant death data and fetal death data files from the National Vital Statistics System of the United States for the years 2003, 2004, and 2005. These publicly available data files, compiled by the U.S. National Center for Health Statistics (NCHS), include information from the birth certificate on maternal socio-demographic characteristics, birth weight, period of gestation, plurality, and live birth order and is linked to information from the infant death certificate. The fetal death files include information from all reports of fetal deaths but do not include the cause of stillbirth [23]. In the United States, states vary in their definitions and reporting requirements for stillbirth [3]. Most states report fetal deaths at 20 weeks or more of gestation and/or 350 grams in birth weight. However, a few states report fetal deaths for all periods of gestation [3]. The most recently available fetal death data file is for 2005.

In the United States, gestational age (GA) is usually calculated from the first day of the mother’s last menstrual period (LMP). It has been shown that gestational age derived from the LMP estimate is prone to error, especially for postterm dates [23-25]. The clinical estimate of gestation has also been recorded since 1989. The clinical estimate is based on the clinician’s best estimate, including menstrual history, physical findings, laboratory values, and (if available) sonography [24]. Recent evidence suggests that the clinical estimate provides rates of preterm birth, postterm birth, and GA-specific rates and relative risks of adverse pregnancy outcomes that are more consistent with those reported in other countries [26,27]. In this study, therefore, our analyses are based on the clinical estimate of gestational age. California does not report the clinical estimate and was therefore excluded from the study.

We restricted our primary analysis to singleton births 24–36 completed weeks of gestation. We also excluded implausibly high or low birth weights at given gestational ages from our study sample, based on Table 1 in Alexander et al.[28] The proportions of live births and stillbirths excluded were 0.6% (n=5,242) and 3.4% (n=826), respectively. Over 65% of excluded stillbirths weighted <350 g, which most states did not report [3]. Our study sample comprised a total of 902,491 preterm births, 23,258 (2.6%) of which were stillbirths.

**Table 1:** Mean birth weight-for-gestational-age z-scores and percent of mean live birth weight at very, moderate, late preterm, and term gestation, Swedish live births and stillbirths, 1992-2001

	**Mean birth weight-for-gestational-age z-score**			**P-value**
	**Live births (n=943,274)**	**Stillbirths (n=3,096)**		
**Preterm** (n=46,531)	−0.29	−1.41		<0.001
		Intrapartum	Antepartum	
		−1.38	−1.41	0.249
28-31 weeks (n=4,783)	−0.96	−1.68	−1.87	
32-33 weeks (n=5,963)	−0.60	−1.14	−1.41	
34-36 weeks (n=35,785)	−0.16	−1.33	−1.01	
**Term** (n=899,812)	+0.03	−0.55		<0.001
		Intrapartum	Antepartum	
		−0.05	−0.59	<0.001
	**Percent of mean live birth weight**			
**Preterm**	100 (ref)	89.2		<0.001
		Intrapartum	Antepartum	
		89.0	88.9	0.283
28-31 weeks	100 (ref)	90.8	87.9	
32-33 weeks	100 (ref)	92.3	89.3	
34-36 weeks	100 (ref)	86.2	89.6	
**Term**	100 (ref)	92.7		<0.001
		Intrapartum	Antepartum	
		98.9	92.2	<0.001

### Swedish births

To guard against potential misclassification of GA in the US birth cohort [24,25], we also analyzed data from the Swedish Medical Birth Register (1992–2001). These data are publicly available for Swedish researchers, including one of the study authors (Professor Cnattingius). The accuracy of gestational age (GA), birth weight, and stillbirth recorded in the Register has been previously validated [29]. In Sweden, gestational age is usually based on a second-trimester ultrasound estimate; otherwise, information on the last menstrual period was used. In Sweden, all women since 1990 are offered an ultrasonic scan performed no later than 18 completed weeks of gestation, and 95% of the women accept this offer [4,29]. As noted earlier, the Swedish Medical Birth Register reported (until recently) only stillbirths with GA ≥28 completed weeks. Further details on the Swedish Medical Birth Register and the study sample have been described elsewhere [15,30,31]. The Swedish data comprised a total of 46,531 preterm births, 1,450 (3.1%) of which were stillbirths. The Swedish data also classify stillbirths as antepartum vs intrapartum, which allowed us to compare the preterm birth weight between these two categories. We further compared birth weight between antepartum and intrapartum stillbirth at term gestation (37–42 weeks), including 899,812 term births, 1,464 (0.2%) of which were stillbirths.

### Construction of fetal growth standard

To evaluate fetal growth, we used estimated fetal weight as the reference standard, rather than the observed birth weight, since the latter is known to be suboptimal at preterm GAs [21,22]. Internal population-based fetal weight standards (separate standards for U.S. and Swedish births) were calculated based on Hadlock’s formula[32] relating the intrauterine (ultrasound) estimated fetal weight (EFW) to gestational age in weeks: log (EFW) = 0.578 + 0.332×GA – 0.00354×GA^2^, which predicts a mean birth weight of 3619 g at 280 days. Hadlock’s formula assumes proportional fetal growth throughout pregnancy and models fetal growth trajectory “by dividing each daily value predicted by this formula by the 280-day value and fitting a third-degree polynomial of gestational age”, which yields the following “proportionality equation” for GAs of 24–39 weeks [33]: % EFW = 299.1 – 31.85 × GA + 1.094 × GA^2^ – 0.01055 × GA^3^.

The latter equation can then be used for any estimated 280-day birth weight to estimate fetal weight in 24–39 weeks of gestational age. Sex-specific birth weight z-scores for 40 weeks of gestation were first calculated based on the study sample, and the sex-specific weight-for-gestational-age z-scores were then extrapolated backward by the proportionality equation. Further details have been described in previous studies [15,30,31]. However, the proportionality equation does not fit well outside the range of 24–39 weeks. In fact, the minimum value for this equation is at 21 weeks, and thus the fetal weight predicted by this formula at 20 weeks is actually higher than that predicted at 21 weeks. For weeks 41 and 42, observed birth weight was used (Swedish births), since birth weight and fetal weight are similar at late GAs [30,31].

### Statistical analysis

The primary outcomes were birth weight and birth weight-for-gestational-age z-score. We compared mean birth weights and birth weight-for-gestational-age z-scores at preterm gestations between singleton stillbirths and live births at 24–36 weeks of gestation from the U.S. and at 28–36 weeks from Sweden. We further compared birth weight-for-gestational-age z-scores between antepartum and intrapartum Swedish stillbirths both at preterm and term gestations. For all these comparisons, we used two-sample t-test. At each completed week of gestation, stillbirth weight was also expressed as a percentage of the mean (POM) of live birth weights at the same gestation; the mean of these percentages, with its 95% confidence interval (CI), was then calculated. We also carried out race/ethnicity-specific analyses of non-Hispanic Whites, non-Hispanic Blacks, and Hispanics, from the U.S. and compared birth weights among antepartum and intrapartum stillbirths from Sweden. Finally, as a sensitivity analysis, we excluded from the U.S. birth cohort all births diagnosed with congenital anomalies at birth and compared birth weight between preterm live births and stillbirths. All analyses were carried out using SAS version 9.2 (SAS Institute Inc., Cary, North Carolina, USA).

## Results

**Figure 1:**
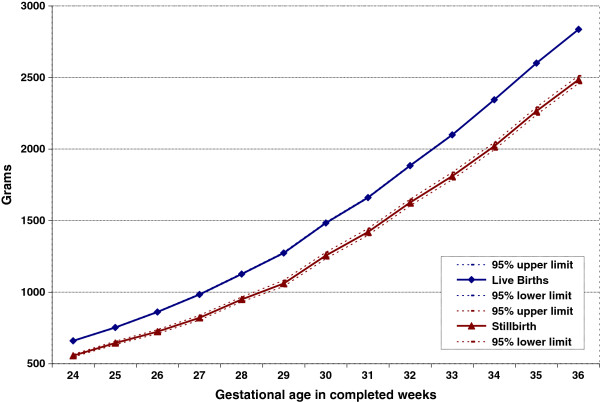
Mean birth weight in preterm live births vs stillbirths, U.S. births 2003–2005.

**Figure 2:**
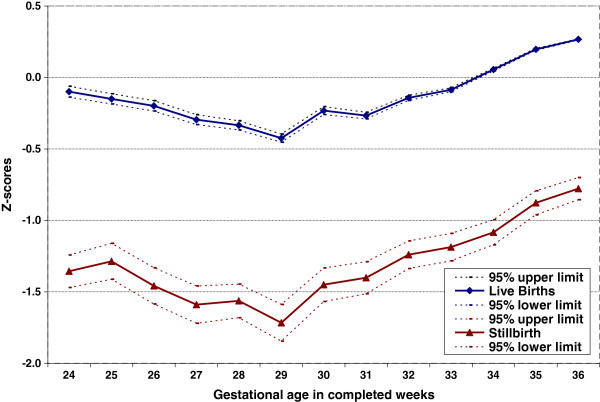
Preterm live birth and stillbirth weight-for-gestational-age z-scores, U.S. births 2003–2005.

**Figure 3:**
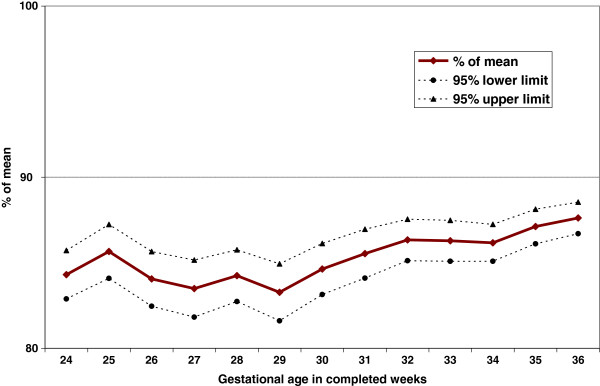
Preterm stillbirth weights expressed as percent of the mean (POM) live birth weight, U.S. births 2003–2005.

Figure 1 shows the mean birth weight with 95% CI, Figure 2 the weight-for-gestational-age z-scores with 95% CI, and Figure 3 the percents of the mean (POM) live birth weight for live births and stillbirths with 95% CI at each preterm GA in the United States from 2003 to 2005. Compared to live births, stillbirth weights were lower at all GAs; the difference increased with gestational age. On average, stillbirths weighed 1,013 g less than live births at preterm gestations. Similarly, stillbirth z-scores were lower than those for live births at all preterm GAs. At 24–33 weeks, live birth z-scores were slightly below 0 (the reference), while at 34–36 weeks, live birth z-scores were slightly above 0. However, stillbirth z-scores were at least one standard deviation lower than live birth z-scores at all GAs between 24 and 36 weeks; the average z-score was −1.57, i.e., more than 1.5 SD below the reference. Moreover, stillbirth weight POMs were at least 10% lower than those of live births at all preterm GAs. All of these differences were highly statistically significant (p<0.001).

We observed similar patterns in birth weight and z-score differences between live births and stillbirths in race/ethnicity-specific analyses of U.S. non-Hispanic Whites, non-Hispanic Blacks, and Hispanics (data not shown) as we did in the overall U.S. study sample. After excluding all births diagnosed with congenital anomalies at birth, we observed a similar patterns of birth weight and z-score differences between preterm live births and stillbirths (data not shown), although the average difference in birth weight decreased from 1,013 to 876 g.

Table 1 compares the weight-for-gestational-age z-scores between antepartum and intrapartum stillbirths and POMs from Sweden at very preterm (28–31 weeks), moderate preterm (32–33 weeks), late preterm (34–36 weeks), and term (37–42 weeks) GAs. At preterm GAs, the differences in z-scores and POMs between antepartum and interpartum stillbirths were not statistically significant, but (as observed in the U.S. data) the differences between live births and stillbirths were highly significant (P < 0.001). In contrast, the z-scores and POMs for antepartum stillbirths at term were significantly lower than for intrapartum stillbirths.

## Discussion

Average birth weights are known to be lower than intrauterine (fetal) weights early in pregnancy [21,22], strongly suggesting that preterm births are undergrown relative to fetuses who remain in utero at the same GA. Birth weights observed at preterm gestational ages are thus “abnormal” overall. The differences observed in Swedish births (Table 1) between preterm live birth weights and estimated intrauterine fetal weights were similar to those reported by Secher et al. [21] and Hutcheon et al. [22]. At 32 weeks, Hutcheon et al. estimated that the median live birth weight is 120 g (i.e., about 0.5 SD) lower than the median fetal weight.

As shown in our study, stillbirth weights were at least one SD below live birth weights at preterm GAs. The fact that stillbirths weigh less at delivery than live births at the same GA has been previously documented [34,35]. Previous studies have also reported that among stillbirths whose time of death is known, most are delivered within 24 hours of fetal death and that the median interval from confirmation of cardiac activity until documentation of death is 7 hours [12-14]. The fetus may lose weight in utero if the death occurs several days or weeks before delivery [3,35]. This is not likely for most stillbirths, however, since the delay between death and delivery is often substantially lower [12-14]. The difference we observed between live births and stillbirths at preterm gestations might be due to the larger proportion of congenital anomalies in preterm stillbirths than among preterm live births [36]. After excluding all births with reported congenital anomalies, however, preterm stillbirths continued to weigh substantially less than preterm live births.

In our analysis of the Swedish data, we were surprised not to observe significant birth weight differences between antepartum and intrapartum stillbirths at preterm GAs. This result does not agree with previous reports by Chard [35] and Alberman et al. [34] Alberman et al., however, did not adjust birth weights for gestational age, and antepartum stillbirth gestational ages averaged 2 weeks lower than those of intrapartum stillbirths [34], while Chard included only stillbirths with GAs 24–32 weeks [35]. The absence of difference in weight between antepartum and intrapartum stillbirths should perhaps not be so surprising at preterm gestations. Why would an intrapartum stillbirth occur at early gestation unless it was seriously compromised before going into labour? In contrast, at GAs of 37–42 weeks, we observed a large difference in weight between antepartum and intrapartum stillbirths but no difference in weight between intrapartum stillbirths and live births.

Assuming little or no weight loss among most antepartum stillbirths from death to delivery, preterm stillbirths appear to be more growth-restricted than live births at the same GA. It has been shown that size at birth is affected by growth velocity early in gestation and that growth restriction diagnosed at delivery is preceded by slower first- and early second-trimester fetal growth [37,38]. As shown in Figure 1, the difference in birth weight between live births and stillbirth increased with advancing GA, suggesting that fetuses who subsequently died in utero grew more slowly before their death, i.e., that fetal growth restriction in stillbirths is a cumulative process.

An important limitation of our study is the lack of a good ultrasound-based fetal weight reference at preterm gestations, which would improve estimation of the difference between the birth weights of live births and the fetal weights of their counterparts who remain in utero. Hadlock’s fetal growth curve [32], based on a modest number of White fetuses from the 1980s, does not appear to fit well with our study sample (U.S. cohort), because birth weight-for-gestational-age z-scores among preterm live births were not substantially below 0 at most preterm GAs, as has been observed in previous studies [21,22]. Another limitation is the lack of information on the precise timing of fetal death; we are thus unable to identify preterm fetuses who died long before the time of diagnosis. This is in particularly true for very preterm stillbirths, owing to less frequent prenatal care visits at early gestations. Furthermore, lack of information on the causes of stillbirths prevents us from exploring the relation between stillbirth weights and causes, and thus the relative weight deficits of stillbirths that are “unexplained.” Finally, the data used in this study are fairly old; the latest U.S. data available for fetal death are from the 2005. There is no reason to suspect, however, that the difference in birth weight between preterm live births and stillbirths would have changed in more recent years.

Our study is further limited by the use of nation-wide vital statistics databases, in which coding errors are known to occur [39,40]. In particular, inaccurate estimation of gestational age in the U.S. has been widely reported and discussed [24,25]. However, our sensitivity analysis based on the Swedish data, in which gestational age is primarily based on second-trimester ultrasound estimates and has been well-validated [29], solidifies our findings. Reporting of fetal deaths is not uniform across the states; a few states report fetal deaths for all periods of gestation and for birth weight <350 g [3]. The use of Table 1 in Alexander et al's excluded mostly extremely low birth weight (65% of the excluded stillbirths were <350 g and nearly 3/4 were < 500 g) due to such non-uniform reporting and thus caused unbalanced exclusion between live births and stillbirths. Without the exclusion, the difference in birth weight between live births and stillbirths at preterm would have been larger. The exclusion of California due to lack of data on clinical estimate of gestation age also limits the generalizability of our study. The proportion of births in California is about 13% of the total U.S. births. Based on our previous study of birth weight trends at term [41], in which menstrual estimates of gestation were used in a sensitivity analysis, such an exclusion is likely to have little impact on our results.

Many preterm births are growth-restricted. Our study suggests that growth restriction is a cumulative process and that those fetuses who subsequently die preterm in utero grow even more slowly then those born alive at the same GA. Reduced placental blood supply and the consequent reduction in delivery of oxygen and nutrients to developing fetuses may both restrict fetal growth early in gestation and increase the risk of subsequent stillbirth. Suboptimal placental function and poor growth early in pregnancy limit fetal growth for the remainder of pregnancy. Early and routine ultrasound scans to monitor fetal size and growth during pregnancy might theoretically reduce the risk of stillbirth. This is perhaps the most important clinical implication of our findings. Before implementing routine clinical monitoring, however, an improved intrauterine fetal weight reference is required. Large-scale NIH- (U.S. National Institutes of Health) and Gates Foundation-funded studies of ultrasound-based fetal size assessment currently under way should provide much-needed data on normal fetal growth patterns. Future research should attempt to understand the pathophysiological mechanisms underlying growth restriction, as a basis for developing and testing preventive and therapeutic interventions to reduce stillbirth risk.

## Conclusions

Birth weights observed at preterm gestation are abnormal, but preterm stillbirths appear to be more growth-restricted than preterm live birth. Similar birth weights among ante- and intrapartum preterm stillbirths suggest serious fetal compromise before the onset of labor.

## Competing interests

We have declared no conflict of interest.

## Authors’ contributions

Dr. XZ designed the study, performed the statistical analyses, and wrote the first draft of the manuscript. Drs. KSJ, SC, and MSK all contributed to the study design, interpretation of results, and manuscript revisions. All authors read and approved the final manuscript.

## Pre-publication history

The pre-publication history for this paper can be accessed here:

http://www.biomedcentral.com/1471-2393/12/119/prepub
